# Computer-Aided Detection for Pancreatic Cancer Diagnosis: Radiological Challenges and Future Directions

**DOI:** 10.3390/jcm12134209

**Published:** 2023-06-22

**Authors:** Mark Ramaekers, Christiaan G. A. Viviers, Boris V. Janssen, Terese A. E. Hellström, Lotte Ewals, Kasper van der Wulp, Joost Nederend, Igor Jacobs, Jon R. Pluyter, Dimitrios Mavroeidis, Fons van der Sommen, Marc G. Besselink, Misha D. P. Luyer

**Affiliations:** 1 Department of Surgery, Catharina Cancer Institute, Catharina Hospital Eindhoven, 5623 EJ Eindhoven, The Netherlands; misha.luyer@catharinaziekenhuis.nl; 2 Department of Electrical Engineering, Eindhoven University of Technology, 5612 AZ Eindhoven, The Netherlands; c.g.a.viviers@tue.nl (C.G.A.V.); t.a.e.hellstrom@tue.nl (T.A.E.H.); fvdsommen@tue.nl (F.v.d.S.); 3 Department of Surgery, Amsterdam UMC, University of Amsterdam, 1105 AZ Amsterdam, The Netherlands; b.v.janssen@amsterdamumc.nl (B.V.J.); m.g.besselink@amsterdamumc.nl (M.G.B.); 4 Cancer Center Amsterdam, 1081 HV Amsterdam, The Netherlands; 5 Department of Radiology, Catharina Cancer Institute, Catharina Hospital Eindhoven, 5623 EJ Eindhoven, The Netherlands; lotte.ewals@catharinaziekenhuis.nl (L.E.); kasper.vd.wulp@catharinaziekenhuis.nl (K.v.d.W.); joost.nederend@catharinaziekenhuis.nl (J.N.); 6 Department of Hospital Services and Informatics, Philips Research, 5656 AE Eindhoven, The Netherlands; igor.jacobs@philips.com; 7 Department of Experience Design, Philips Design, 5656 AE Eindhoven, The Netherlands; jon.pluyter@philips.com; 8 Department of Data Science, Philips Research, 5656 AE Eindhoven, The Netherlands; dimitrios.mavroeidis@philips.com

**Keywords:** pancreatic ductal adenocarcinoma, diagnostics, artificial intelligence, computer-aided detection, radiological imaging, clinical implementation

## Abstract

Radiological imaging plays a crucial role in the detection and treatment of pancreatic ductal adenocarcinoma (PDAC). However, there are several challenges associated with the use of these techniques in daily clinical practice. Determination of the presence or absence of cancer using radiological imaging is difficult and requires specific expertise, especially after neoadjuvant therapy. Early detection and characterization of tumors would potentially increase the number of patients who are eligible for curative treatment. Over the last decades, artificial intelligence (AI)-based computer-aided detection (CAD) has rapidly evolved as a means for improving the radiological detection of cancer and the assessment of the extent of disease. Although the results of AI applications seem promising, widespread adoption in clinical practice has not taken place. This narrative review provides an overview of current radiological CAD systems in pancreatic cancer, highlights challenges that are pertinent to clinical practice, and discusses potential solutions for these challenges.

## 1. Introduction

Pancreatic ductal adenocarcinoma (PDAC) is one of the leading causes of cancer-related deaths worldwide, with a dismal prognosis and an overall 5-year survival rate of only 9% [1,2]. Although pancreatic cancer treatment has improved over the past years through centralization and optimization of treatment strategies, overall survival has not significantly improved [3,4]. Pancreatic cancer often causes only a few non-specific symptoms before it develops into advanced stages of disease. The most commonly presented symptoms in patients with PDAC are pain, jaundice, steatorrhea, and weight loss [5]. As a result, more than 75% of the patients present with irresectable or metastatic disease [6,7]. Therefore, early detection of pancreatic tumors holds significant promise by enabling potential curative treatment [8]. Subsequently, characterization of pancreatic tumors is important in order to tailor specific treatments, determine surgical resectability, and identify each patient for curative treatment as well as possible.

Radiological imaging modalities such as computed tomography (CT) and magnetic resonance imaging (MRI) are key in providing information on the presence of disease and the relation to vessels surrounding the pancreas, which determines the resectability [9]. Standardized resectability criteria are used to tailor the need for neoadjuvant therapy and to select patients for a minimally invasive approach [10,11,12]. However, determining resectability, especially after neoadjuvant therapy, is extremely difficult and mostly inaccurate at this time [13,14]. Tumor regression after neoadjuvant treatment is rarely visible on CT, and the extent of vascular involvement tends to be overestimated [15].

Artificial intelligence (AI) offers a unique opportunity to improve the early detection and characterization of pancreatic cancer. Over the past decades, deep learning-based algorithms have been developed that can provide pixel-level segmentations of relevant anatomy. Deep learning refers to the use of a neural network with multiple layers suited for recognizing features from input data. However, implementation in healthcare proceeds slowly, and the majority of AI solutions remain within the testing and prototyping phases [16,17].

Here, we provide an overview of current AI applications in the radiological detection of pancreatic cancer, highlight challenges in clinical practice, elaborate on the current state of computer-aided detection, and discuss future perspectives.

## 2. Rationale for Early Detection of Pancreatic Cancer

### 2.1. Radiological Detection

Pancreatic cancer often goes undetected until it has progressed into an advanced stage, and therefore only a minority of patients are eligible for surgical resection at the time of diagnosis [18]. The initial diagnosis of pancreatic tumors mostly starts with CT imaging. Multi-phase (arterial and portal-venous) contrast-enhanced multi-detector computed tomography (MDCT) is standard practice in evaluating pancreatic cancer and carries a sensitivity of at least 90% in expert hands [19,20,21]. In general, pancreatic tumors appear hypodense compared to normal pancreatic parenchyma. However, there may be other imaging findings predictive of pancreatic cancer as well. The multi-phase protocol provides good spatial resolution and allows proper visualization of relevant structures and vasculature to assess staging and resectability [18].

In addition to CT imaging, MRI allows for successful tumor detection. Despite the soft-tissue contrast, MRI has been shown to be as sensitive as CT in diagnosing and staging pancreatic cancer, with a sensitivity of 89% [22]. While MRI is not widely used as an initial imaging technique due to cost and availability, there are several specific situations where it is preferred. The soft tissue contrast of MRI may be beneficial in the recognition of small tumors and the characterization of indeterminate liver lesions [23].

Lastly, endoscopic ultrasound (EUS) is often used to discriminate between PDAC and other pancreatic diseases. EUS offers several advantages for pancreatic tumor detection, including its ability to provide detailed imaging of the pancreas and its surrounding structures, as well as the possibility of obtaining tissue samples for histological analysis [24]. EUS has shown high sensitivity in detecting pancreatic cancer, and several studies have reported a sensitivity ranging from 80% to 95% [25]. However, the sensitivity of EUS can be influenced by various factors, including the size and location of the tumor and the experience of the endoscopist performing the procedure.

### 2.2. Restrictions for Early Detection and Characterization of Pancreatic Cancer

Early detection and characterization of pancreatic tumors represent one of the most promising strategies to improve the prognosis and overall survival of pancreatic cancer patients [26,27]. This can be attributed to several factors. First, patients diagnosed in early disease stages often have smaller tumors (<2.0 cm) with less vascular involvement. Although only a minority of patients (10–15%) are diagnosed in early stages and have stage-1 disease, they present a much higher 3-year survival rate (82%) compared to patients diagnosed in later disease stages where tumors are larger (17%) [28,29]. Second, pancreatic imaging requires specific expertise, and with small and iso-attenuating tumors sometimes being barely visible, the radiologist has to rely on other patterns that might indicate malignant disease. Although CT and MRI generally achieve acceptable sensitivity measures in diagnosing pancreatic cancer, subtle pancreatic changes may be missed on abdominal imaging, especially in asymptomatic patients [30,31]. Radiologists’ sensitivity to detecting small and iso-attenuating PDACs with sizes smaller than 2 cm on CT has been reported to be between 58 and 77% [32]. Lack of expertise may result in delayed recognition and prevent patients from receiving curative treatment. This may even be more pertinent in hospitals without specific pancreatic expertise [33]. Over the years, various studies have reported the presence of visible secondary features prior to actual diagnosis [34,35,36]. Kang et al. demonstrated that secondary signs are present in 88% of the cases. The most common secondary sign was pancreatic duct dilation, and vascular invasion was the most commonly missed [31]. In addition, studies reported that indicative changes of PDAC are visible on imaging 6–18 months prior to actual diagnosis in 50% of patients [35,37]. Third, pancreatic cancer treatment is centralized, which limits expertise to certain hospitals. Previous studies have shown that patients with non-metastasized pancreatic cancer had a greater likelihood of receiving surgical treatment when the diagnosis was established in an expert pancreatic cancer center compared to non-expert hospitals [38]. The centralization of pancreatic surgery may further enhance this discrepancy between expert and non-expert hospitals. Multidisciplinary team meetings may preserve expertise in various treatment techniques; however, patients still need to be identified before expert assessment can be performed.

For the characterization of PDAC, resectability is graded as resectable, borderline resectable, or irresectable based on the contact between the tumor and surrounding vasculature [39]. However, the assessment of resectability based on a CT scan remains challenging with considerable interobserver variability, even among the most experienced clinicians [40,41]. Furthermore, between 4 and 13% of patients with operable pancreatic cancer are found to be unresectable at the time of surgery due to missed metastases or inadequate resectability assessment [42]. Following neoadjuvant treatment, assessment of resectability becomes even more difficult. Tumor regression after neoadjuvant treatment is rarely visible on radiological assessment, and literature shows that the diagnostic performance of radiologists to predict resectability or irresectability on CT is inaccurate [13,15]. As such, clinicians are unable to accurately assess tumor resectability after neoadjuvant treatment. Additionally, the extent of vascular involvement tends to be overestimated, and several studies have indicated considerable variability in resectability assessment between medical experts, which could affect treatment strategies [40,43]. Meanwhile, different strategies of neoadjuvant treatment followed by surgery have been shown to improve overall survival in patients with borderline resectable pancreatic cancer [44,45]. Based on these promising results of neoadjuvant treatment, it is to be expected that neoadjuvant treatment evaluation will become an increasing problem.

## 3. Computer-Aided Detection as a Potential Solution

### 3.1. Pancreatic Cancer Detection Using CAD

Artificial intelligence (AI) has emerged as a powerful tool in medical healthcare, and by analyzing large amounts of medical imaging data, AI algorithms can identify subtle changes in the pancreas that may be missed by human observers. A potential tool that may facilitate the improvement of pancreatic tumor detection and response evaluation is computer-aided detection (CAD) based on artificial intelligence techniques. Over the past decades, deep learning has rapidly advanced the development of CAD methods across various domains, and it is expected that it will continue to act as a driver for technological innovation in the foreseeable future [46]. The first steps towards automated pancreatic tumor detection on CT have been conducted. Overall, two main approaches exist for CAD development: 1. deep learning and 2. radiomics.

In deep learning, artificial neural networks are employed as a machine learning technique, and these models are trained to detect and localize pancreatic cancer based on a large number of annotated examples. In radiomics, large amounts of hand-crafted image features are extracted from digital images, also known as radiomics features. These features then serve as a basis for conventional machine learning models to make predictions about the data. 

In recent years, several studies have demonstrated promising methods and results using AI to detect pancreatic cancer on CT scans. An overview of the most notable results and architectures is summarized in Table 1. Although several studies reported notable results, it is important to consider that some studies only performed binary classification of CT scans, meaning that they only classified tumor presence or absence. To provide patients with adequate treatment, clinicians often require additional results, such as the location of the tumor. Additionally, AI-based tools can be leveraged to distinguish PDAC from auto-immune pancreatitis. Various studies have demonstrated impressive results in distinguishing auto-immune pancreatitis from PDAC, exploiting both deep learning- and radiomics-based approaches [47,48]. Recently, Rigiroli et al. took a first step in investigating whether tumor-related and perivascular CT radiomic features improve preoperative assessment of arterial involvement in patients with surgically proven PDAC. The model showed a sensitivity and specificity of 0.620 and 0.770, respectively, and a better performance compared to the radiologist’s assessment [49]. 

Quantitative MRI techniques such as T1 and T2 image mapping allow for accurate tissue characterization and provide early indicators of biological changes [63]. Additionally, it offers a nonionizing radiation alternative. However, the availability of high-quality MRI data is limited, and literature on the detection of PDAC using MRI is scarce. Kaissis et al. provided extensive work by applying machine learning to MR images to preoperatively predict survival and molecular subtypes in patients with PDAC [64,65,66]. Their survival model achieved impressive results with a sensitivity and specificity of 0.870 and 0.800, respectively, and an area under the curve (AUC) of 0.90 for the prediction of above- and below-median overall survival (OS) [64]. Liang et al. specifically aimed to develop a deep learning algorithm allowing automatic segmentation of gross tumor volume and reported performances, similar to expert radiation oncologists [67]. Over the years, only a few other studies reported MRI-based machine learning models and mainly focused on the identification and characterization of pancreatic abnormalities. An overview of the most notable results is summarized in Table 2.

AI-based algorithms can be integrated with EUS to assist in the interpretation and analysis of the acquired images. By training AI models on large datasets of EUS images, these algorithms can learn to recognize patterns and features indicative of pancreatic tumors. In addition, they can help reduce inter-observer variability and improve the diagnostic consistency of EUS, as endoscopist experience plays an important role in the accuracy of this imaging technique. The added value of AI to discriminate between benign and malignant tissue during EUS has been investigated in a considerable number of studies. Several studies have explored the use of AI in differentiating between PDAC and the normal pancreas, and all models achieved an overall accuracy of at least 90% [72,73,74]. Additionally, several studies focused on differentiating between PDAC and chronic pancreatitis using EUS, with all models accurately predicting PDAC in over 80% of the cases [75,76,77].

Lastly, assessment of vascular involvement and determination of treatment response are still growing problems without accurate assessment tools. Different scoring systems have been proposed to predict vascular involvement and, thus, resectability status in pancreatic cancer patients [78,79]. Given the beneficial effect of neoadjuvant treatment on cancer-specific survival, a recent study developed tumor-vessel interface criteria to predict vascular involvement and resectability in borderline pancreatic cancer patients [80]. The diagnostic performance for predicting vascular involvement was evaluated between 2 readers and showed an AUC for agreement of 0.85–0.88 for arterial invasion and 0.87–0.92 for venous invasion. Furthermore, CT texture analysis for predicting resectability after neoadjuvant treatment has been introduced, providing important information regarding tumor characterization by quantifying tissue heterogeneity and texture coarseness [81,82].

### 3.2. AI and the Use of Biomarkers for Pancreatic Cancer Detection

In addition to imaging, there is ongoing research to identify biomarkers that can aid in the early detection of pancreatic cancer. In contrast to other malignancies, there are few sensitive circulating biomarkers, and currently there is no standardized screening strategy. Several conventional biomarkers have shown promise in the detection of pancreatic cancer. Carbohydrate antigen 19-9 (CA19-9) and carcinoembryonic antigen (CEA) are the most widely used biomarkers for the screening of PDAC [83]. Unfortunately, both biomarkers have been proven to lack sensitivity and specificity for early-stage detection, and they are usually used in monitoring treatment response and detection of recurrent pancreatic cancer [84]. Combined measurements can potentially increase the sensitivity and specificity of biomarkers, as Yang et al. developed a neural network for detecting pancreatic cancer combining multiple tumor markers (CA19-9, CEA, and CA125). In a retrospective analysis of 913 serum specimens, the performance of the neural network was superior to each of the serum tumor markers alone, with an AUC of 0.905 and a diagnostic accuracy of 83.5% [85].

Additionally, other novel biomarkers have been identified that are present in early pancreatic adenocarcinomas, such as microRNAs (miRNAs). MiRNAs are small RNA molecules that regulate gene expression. Specific miRNAs such as miR-21, miR-155, and miR-196a are present in pancreatic fluids, have been identified as dysregulated in pancreatic cancer, and may serve as diagnostic biomarkers [86]. However, they tend to have the same limitations in sensitivity and specificity as conventional biomarkers. An interesting study by Cao et al. used machine learning to identify two diagnostic panels based on miRNA expression in plasma to differentiate between pancreatic cancer and chronic pancreatitis. Using 361 plasma samples from six centers in China, they achieved an accuracy of 83.6% for distinguishing pancreatic cancer from chronic pancreatitis. In addition, they demonstrated that the diagnostic value of both panels was comparable to that of CA19-9 [87].

To date, AI-based algorithms have shown great promise in medical image analysis, including CT scans and MRIs. The integration of multiple biomarkers and AI-based image analysis could enable a more comprehensive evaluation of the disease, facilitating the identification of high-risk individuals and potentially detecting pancreatic cancer at earlier stages when treatment options are more effective. Therefore, the most promising strategy for developing complete screening tests for pancreatic cancer should include a combination of imaging data, biomarkers, and patient data. AI-based algorithms can leverage this combined information to learn patterns and features, potentially leading to improved diagnostic accuracy and comprehensive screening of patients.

### 3.3. Representative Data, Bias, and Confounders

Earlier forms of AI applications have failed to achieve widespread adoption, mainly due to their limited technical performance of these early applications [88,89]. AI’s unique strength is its ability to learn from complex datasets and recognize subtle patterns; however, it also suffers from a host of imperfections, including inapplicability to different populations, bias, and accidentally fitting confounders [16]. 

Most AI models are far from achieving reliable generalizability because building a trustworthy model requires a representative and diverse dataset that resembles the expected target population during use. Models are trained using variable methodologies on different populations with their respective characteristics and are therefore not automatically transferable to other hospitals, as illustrated in a recent study to detect abnormal chest radiographs. At a fixed operating point, specificity varied widely from 0.566 to 1.000 across five independent datasets [90]. Independent local datasets that represent a sample of their own population could be used as supplementary local training datasets to allow adaptation of an existing algorithm before formal testing. For less complicated tasks such as medical image classification, this problem may be less crucial and might be overcome using large, heterogeneous, multicenter datasets [91].

Another major concern is that datasets used to train AI models could have unintended biases present in historical data. Biases include those related to missing data, sample size, underestimation, misclassification, and measurement error. There is also a general concern that biases in the data used by machine learning algorithms may contribute to socioeconomic disparities in healthcare [92]. Algorithms introduced both in medical and non-medical fields already showed some problematic recommendations that reflect biases inherent in the data used for training [93]. The prediction of an offender’s risk of recidivism has shown a tendency for racial discrimination, just as predicting the risk of cardiovascular events in non-white populations [94,95]. Similar biases could inadvertently be built into healthcare algorithms. To reduce such risks, the prediction model risk of bias assessment tool (PROBAST) criteria can be used to assess the risk of bias for prediction model studies [96].

Finally, machine learning algorithms may include confounding factors and spurious correlations to achieve the best possible performance [16]. In this way, the algorithm would draw conclusions based on accidental correlations found in the training data, impairing the algorithm’s ability to generalize to new datasets. Remarkable examples of accidental fitting confounders in healthcare are the presence of a ruler or surgical skin markings, which the algorithm correlated with an increased likelihood of a cancerous skin lesion [97,98]. Therefore, ongoing development is required to understand the specific features and possible biases that are being learned by AI models before adopting them in healthcare.

### 3.4. Acceptance of AI in Daily Workflow

Over the last decades, AI has rapidly become one of the major academic research subjects in medical image interpretation, and the number of AI studies has grown at an exceptional rate. Thousands of AI solutions for radiology have been developed in research labs, and, to date, some of these perform on par or even better than clinicians [99,100,101]. However, a recent study showed that most AI applications remain within the testing and prototyping environment, and surprisingly few applications successfully make their way from the research lab into clinical practice [102]. This may be explained in several ways. 

Most of the current evidence is based on retrospective studies with extensive benchmarking of the algorithm’s performance against experts’ performance [103]. A higher level of evidence is needed to understand the true performance of AI systems when encountering real clinical data. Although some studies provide excellent results, purely focusing on the algorithm’s performance can come at the expense of study quality and the risk of bias. Hence, regulatory guidelines have been proposed by various parties to guarantee patient safety [104,105]. Recently, Van de Sande et al. proposed a step-by-step approach to improve the quality, safety, and transparency of AI research and to improve clinicians understanding of AI technology [102]. 

Another well-recognized cause for this low adoption and actual use is a lack of human-centered AI design. Poor fit into the working lives of healthcare providers and a lack of trust or trustworthiness have been shown to hamper the uptake of AI into daily clinical workflow [16,17]. Physicians often work on tight schedules, and if AI creates additional complexity, physicians will have a hard time embracing new innovations.

In an environment where physicians have to make split-second decisions with potentially far-reaching consequences, trust in new technology is important but may also be fragile. This is especially pertinent as AI poses unique potential trust issues, such as the frequent lack of explainability of its results (the black box) and a tendency to occasionally produce unexpected recommendations. It is essential to advance AI from the lab into a clinical setting while keeping humans (patients and physicians) central to the development and evaluation process. Finally, due to the lack of substantial evidence on the added value of AI applications, the acquisition of funds can be uncertain. 

### 3.5. Discussion for Future Development

AI has grown as a high-potential technology that will shape the future of medical healthcare, and deep learning with convolutional neural networks (CNN), transformers, and diffusion models has rapidly revolutionized the standard for AI applications. However, translation of research techniques into effective clinical deployment presents a new opportunity for clinical, machine learning, and human-computer interaction design research. Considering the current issues, we believe the following aspects will become increasingly important for the further progression of AI in pancreatic cancer detection: 

First, training a deep learning algorithm for complicated tasks requires large amounts of high-quality labeled data. However, due to the relatively low prevalence of pancreatic cancer, this is one of the biggest hurdles hampering the training of AI-based algorithms. The first indications of the potential of AI applications are clearly visible in retrospective research on small, homogeneous datasets, where these systems demonstrated impressive results in the detection of pancreatic tumors. However, datasets are the primary drivers of AI-based algorithms, and collaboration and data sharing between expert centers for pancreatic surgery would provide the opportunity to deal with the scarcity of high-quality labeled datasets.

Second, for proper evaluation of the model, the results should be presented transparently, following clear standards. To avoid misconceptions, it should be clear which metrics were used to evaluate the performance of the AI model. Metrics such as accuracy, sensitivity, specificity, and area under the receiver’s operating characteristics should be described [102,106]. Therefore, clear guidelines can be used following transparent reporting of a multivariable prediction model for individual prognosis or diagnosis (TRIPOD) [107,108]. Additionally, a targeted calculation of the sample size of the test set, as proposed by Riley et al., which is needed to accurately determine the quality of early-stage algorithms, could facilitate an unbiased evaluation [109].

Third, for an algorithm to be of added value in clinical practice, aspects such as reliability, uncertainty, and robustness against variation will become increasingly important. This may require novel approaches that require strong collaboration between clinicians and AI. While detection of cancer is the first essential step, follow-up tasks such as tumor resection planning require extremely detailed segmentations of the tumor and surrounding anatomical structures. Current existing approaches do not yet meet this high accuracy requirement. The current research trend is to design better models to adhere to these shortcomings, while it is possible that there will always be edge cases unaccounted for or different opinions on the correct segmentation. A possible solution to bridge the gap between what is technically possible and what is clinically necessary is interactive AI (IAI), where a user can provide AI with additional input. By implementing IAI, the pattern recognition abilities of AI and the domain knowledge of the clinician can be combined, resulting in more accurate and robust results [110]. This could enable clinicians to achieve highly accurate annotations of the structures of interest and could improve the effectiveness and implementation of AI algorithms in clinical practice.

Fourth, multidimensional radiological techniques, such as PET-CT and PET-MRI, may offer significant added value in combination with AI-based approaches. Through the use of positron emission tomography (PET), these techniques provide complementary quantitative information, such as metabolic activity, perfusion, and microenvironment, to traditional CT and MRI imaging [111]. This allows for a more accurate and comprehensive assessment of pancreatic tumors. AI-based algorithms can leverage this combined information to learn patterns and features, potentially leading to improved diagnostic accuracy [112,113]. Additionally, intelligent 4D imaging on ultra-high-sensitivity PET, such as Large Axial Field of View (LAFOV) PET, could enhance the detection of small lesions and improve image quality. 4D imaging refers to the acquisition of imaging data over time, allowing for the assessment of dynamic processes within the body [114]. Potentially, AI algorithms can analyze and interpret the data obtained from LAFOV PET, which could enable a more accurate assessment of tumor behavior or prediction of disease progression. Integration of AI with multidimensional imaging techniques, including 4D imaging, may also aid in the development of personalized treatment strategies, as it can provide valuable insights into tumor biology, heterogeneity, and response to therapy.

Lastly, the development and implementation of AI require an interdisciplinary approach between technological and medical partners. A strong collaboration between medical doctors and engineers is necessary to create a common understanding of the possibilities and limitations of AI. After all, it has already been shown that humans assisted by AI performed better than either alone in a study of diabetic retinopathy screening [115]. Understanding human-algorithm interactions and the importance of human-centered AI will be critical to the future adoption of AI applications. The engine of the AI model, as well as the user interface (UI) through which the physician and AI interact, must be optimized for this team’s performance. Choices in AI development and UI design influence how physicians work with AI to leverage the collective intelligence of the physician and the AI [116]. Additionally, facilitating smooth integration will be crucial for clinical adoption. Medical practitioners are often hesitant to adopt new technology that interrupts their traditional practice patterns [117]. The additional time that a new technology requires to be implemented is a significant barrier for clinicians’ user acceptance since their high workloads prevent them from taking time for an adequate implementation.

## 4. Conclusions

AI is a valuable approach to overcome the current challenges in the diagnosis and optimal treatment of pancreatic cancer. By facilitating applications for early detection, assessing resectability, and determining treatment response to neoadjuvant therapy, AI can revolutionize pancreatic cancer detection. However, AI in pancreatic cancer is still in its infancy, and more collaborations between technical expertise and clinical practice are needed to design the applications that are going to improve diagnostic accuracy. In order to progress, future research should focus on three key challenges: (1) the use of representative heterogeneous datasets; (2) the transparent presentation of results; and (3) understanding the importance of human-centered AI. Over the past decades, the exponential increase in the amount of visual data and computational power has enabled AI to demonstrate its endless capabilities. Clinical adoption of AI will be catalyzed by high-quality prospective studies and optimal human-AI interaction. 

## Figures and Tables

**Table 1:** Studies investigating artificial intelligence-based pancreatic ductal adenocarcinoma detection using CT.

Author (Year)	Aim of Model	Type of Model	Dataset (*n*)	Sensitivity (%)	Specificity (%)	AUC	Accuracy (%)
Chu et al. (2019) [50]	Detection of PDAC	3D U-Net (CNN)	156 PDAC 300 Control	94.1	98.5	N.A.	N.A.
Liu et al. (2019) [51]	Detection of pancreatic cancer	Faster R-CNN	338 Pancreatic cancer patients	N.A.	N.A.	0.963	N.A.
Zhu et al. (2019) [52]	Detection of PDAC	3D U-Net (CNN)	136 PDAC 303 Control	94.1	98.5	N.A.	57.3
Chu et al. (2019) [53]	Detection of PDAC	Radiomics	190 PDAC 190 Control	100	98.5	0.999	99.2
Liu et al. (2020) [54]	Detection of pancreatic cancer	VGG-CNN	370 Pancreatic cancer 320 Control	97.3	100	0.997	98.6
Zhang et al. (2020) [55]	Detection of pancreatic cancer	Custom CNN Feature Pyramid	2890 CT images	83.7	91.7	0.945	90.2
Ma et al. (2020) [56]	Detection of pancreatic cancer	Encoder only CNN	222 PDAC 190 Control	91.6	98.3	0.965	95.5
Si et al. (2021) [57]	Detection of pancreatic cancer	ResNet and U-Net (CNN)	319 patients	86.8	69.5	0.872	87.6
Qiu et al. (2021) [58]	Diagnosis analysis of PDAC	Radiomics	312 patients	N.A.	N.A.	0.880	81.2
Ebrahimian et al. (2022) [59]	Differentiating malignant and benign pancreatic lesions	Radiomics	103 patients	84.0	95.0	0.990	0.91
Viviers et al. (2022) [60]	Detection of PDAC	3D U-Net (CNN)	99 PDAC 97 Control	99.0	99.0	N.A.	N.A.
Alves et al. (2022) [61]	Detection of PDAC	3D U-Net (CNN)	119 PDAC 123 Control	N.A.	N.A.	0.909	N.A.
Chen et al. (2023) [62]	Detection of pancreatic cancer	3D U-Net (CNN)	546 pancreatic cancer patients 733 control	89.7	92.8	0.950	N.A.

Legend: CT, computed tomography; AUC, area under the curve; CNN, convolutional neural network; VGG, visual geometry group; PDAC, pancreatic ductal adenocarcinoma.

**Table 2:** Studies investigating artificial intelligence-based pancreatic cancer diagnosis using MRI.

Author (Year)	Aim of Model	Type of Model	Dataset (*n*)	Sensitivity (%)	Specificity (%)	AUC	Accuracy (%)
Gao et al. (2019) [68]	Histopathological WHO grade prediction of pNET	CNN Encoder	96 patients	N.A.	N.A.	0.885	81.1
Corral et al. (2019) [69]	Identify neoplasia in IPMN	CNN feature representation + SVM	139 cases	75.0	78.0	0.780	N.A.
Kiassis et al. (2019) [64]	Survival prediction of PDAC	Random Forest Machine learning	102 PDAC patients	87.0	80.0	0.900	N.A.
Kiassis et al. (2019) [65]	Molecular subtype prediction of PDAC	Radiomics	55 PDAC patients	90.0	92.0	0.930	N.A.
Gao et al. (2020) [70]	Differentiate pancreatic diseases	Inception v4 (CNN)	398 patients	N.A.	N.A.	0.864	76.8
Kiassis et al. (2020) [66]	Molecular subtype prediction of PDAC	Random Forest Machine learning	207 PDAC patients	84.0	92.0	0.930	N.A.
Deng et al. (2021) [71]	Differentiate PDAC from MFP	Radiomics	52 PDAC patients 13 MFP patients	N.A.	N.A.	0.945	79.5

Legend: MRI, magnetic resonance imaging; AUC, area under the curve; WHO, world health organization; CNN, convolutional neural network; PDAC, pancreatic ductal adenocarcinoma; IPMN, intraductal papillary mucinous neoplasm; MFP, mass-forming pancreatitis; pNET, pancreatic neuroendocrine tumor.

## Data Availability

The data presented in this study are available on request from the corresponding author.
